# Imaging Flow Cytometry and Convolutional Neural Network-Based Classification Enable Discrimination of Hematopoietic and Leukemic Stem Cells in Acute Myeloid Leukemia

**DOI:** 10.3390/ijms25126465

**Published:** 2024-06-12

**Authors:** Trine Engelbrecht Hybel, Sofie Hesselberg Jensen, Matthew A. Rodrigues, Thomas Engelbrecht Hybel, Maya Nautrup Pedersen, Signe Håkansson Qvick, Marie Hairing Enemark, Marie Bill, Carina Agerbo Rosenberg, Maja Ludvigsen

**Affiliations:** 1 Department of Hematology, Aarhus University Hospital, 8200 Aarhus N, Denmark; trihyb@rm.dk (T.E.H.); mariem@rm.dk (M.H.E.);; 2 Department of Clinical Medicine, Aarhus University, 8200 Aarhus N, Denmark; 3 Amnis Flow Cytometry, Cytek Biosciences, Seattle, WA 98119, USA

**Keywords:** imaging flow cytometry, artificial intelligence, deep learning, convolutional neural network, hematopoietic stem cells, leukemic stem cells, acute myeloid leukemia

## Abstract

Acute myeloid leukemia (AML) is a heterogenous blood cancer with a dismal prognosis. It emanates from leukemic stem cells (LSCs) arising from the genetic transformation of hematopoietic stem cells (HSCs). LSCs hold prognostic value, but their molecular and immunophenotypic heterogeneity poses challenges: there is no single marker for identifying all LSCs across AML samples. We hypothesized that imaging flow cytometry (IFC) paired with artificial intelligence-driven image analysis could visually distinguish LSCs from HSCs based solely on morphology. Initially, a seven-color IFC panel was employed to immunophenotypically identify LSCs and HSCs in bone marrow samples from five AML patients and ten healthy donors, respectively. Next, we developed convolutional neural network (CNN) models for HSC-LSC discrimination using brightfield (BF), side scatter (SSC), and DNA images. Classification using only BF images achieved 86.96% accuracy, indicating significant morphological differences. Accuracy increased to 93.42% when combining BF with DNA images, highlighting differences in nuclear morphology, although DNA images alone were inadequate for accurate HSC-LSC discrimination. Model development using SSC images revealed minor granularity differences. Performance metrics varied substantially between AML patients, indicating considerable morphologic variations among LSCs. Overall, we demonstrate proof-of-concept results for accurate CNN-based HSC-LSC differentiation, instigating the development of a novel technique within AML monitoring.

## 1. Introduction

Acute myeloid leukemia (AML) is a highly heterogenous blood cancer with a dismal prognosis. An incidence rate of 1.61/100,000 and mortality rate of 1.22/100,000 were reported globally in 2019 [1]. The malignancy originates from genetic aberrancies in hematopoietic stem cells (HSCs) in the bone marrow (BM), causing the formation of a pool of rare leukemic stem cells (LSCs) with survival advantages, increased proliferation potential, and blocked differentiation. Healthy hematopoiesis is suppressed, and LSCs give rise to leukemic blasts that proliferate rapidly, accumulate in the BM and peripheral blood (PB), and cause clinical manifestations of BM failure [2,3,4,5]. Untreated AML will usually result in death within months of diagnosis due to bleeding or infection. Consequently, rapid classification, prognostication, and treatment initiation remain crucial [6,7,8]. Estimated 5-year survival rates vary significantly between age groups, being approximately 50% in younger adults (<60 years) and merely around 10% in the elderly [9].

Even though induction chemotherapy can induce remission in up to 60–80% of younger patients, relapse rates are high [5,10,11,12,13]. Relapses are believed to emanate from residual leukemic cells, consisting of a small fraction of blasts and/or quiescent LSCs, which is referred to as minimal residual disease (MRD) [2,5]. Importantly, the presence of MRD after induction chemotherapy is strongly associated with an increased risk of relapse and death, and for intermediate risk patients, MRD-guided clinical decision making is currently employed to decide whether or not to consolidate with allogeneic BM transplantation [14,15,16]. As MRD remains below the detection threshold of cytomorphological evaluation, assessment using other standardized and sensitive methods is essential to evaluate treatment response and predict impending relapse. The most extensively studied techniques include multiparameter flow cytometry (MFC) and reverse transcription quantitative polymerase chain reaction (RT-qPCR). The latter is applicable to follow a molecular marker in approximately 60% of cases and includes detection of fusion transcripts, mutations, and overexpression of specific genes. For other AML patients, the use of MFC is recommended. This entails monitoring a pre-determined diagnostic leukemia-associated immunophenotype (LAIP) through a “different-from-normal” (DfN) approach, which involves comparing leukemic immunophenotypes to those observed in healthy cells. In this way, MFC is useful to track and identify abnormal immunophenotypes in approximately 90% of cases. Additionally, digital droplet PCR and next-generation sequencing (NGS) are currently under investigation for detection of MRD [10,16,17,18,19]. Despite the usefulness of current MRD estimates, some MRD-negative patients may still experience relapse due to the leukemia remaining below the detection threshold [16]. Of note, LSCs are believed to be enriched in the MRD cell population, and consequently, surveillance of LSCs during treatment holds promising prognostic information [20]. In fact, inclusion of immunophenotypic detection of LSCs by MFC into current MRD techniques is associated with higher sensitivity and fewer false-negative results [14,21,22]. Nevertheless, LSC monitoring is currently not included in the diagnostic workup for AML patients, although it is recommended in clinical studies [16,22].

Importantly, current MRD techniques focus on the total leukemic burden, including both leukemic blasts and LSCs. As LSCs are ultimately responsible for propagating AML, accurate detection of LSCs and distinguishment from their healthy HSC counterparts would greatly improve therapeutics and disease monitoring [22]. Numerous immunophenotypic markers have been shown to be differentially expressed in LSCs and their healthy HSC counterparts, including CLEC12A, CD7, CD123, TIM3, CD70, and CD9 [4,5,15,23,24,25]. In this regard, most studies have focused on CD34^+^ AML, where LSCs primarily co-reside with healthy HSCs within the CD34^+^CD38^−^ compartment [26,27]. Importantly, considerable immunophenotypic heterogeneity exists, and no single marker identifies all LSCs across AML samples [5,28]. For example, Petersen et al. detected 50 LSC subsets showing 41 different immunophenotypic profiles within BM samples from 38 pediatric AML patients [29,30]. Also, the absence of an LSC marker on AML stem cells does not necessarily equal absence of disease, as shown by Bill et al. demonstrating disease-related aberrancies within CLEC12A^−^ stem cell subsets from AML cases [31]. Healthy HSCs from normal BM, on the other hand, are always CLEC12A^−^ [32]. While most studies have been performed using pre-treatment samples, markers may vary throughout the disease course [2,5,28,33]. These substantial immunophenotypic variations between and within each case considerably challenge the clinical applicability of LSC monitoring and emphasize the need for alternative discriminative parameters to reliably detect LSCs. Terwijn et al. revealed substantially higher forward scatter (FSC) and side scatter (SSC) properties among marker-positive LSCs compared to HSCs from the CD34^+^CD38^−^ stem cell compartment [4,22], indicating inherent morphological variations between the cell types. Based on this, as an alternative to immunophenotypic detection, we investigate a novel method for identifying LSCs using differences in morphology detected by imaging flow cytometry (IFC) combined with artificial intelligence (AI)-based image analysis.

The IFC technology is a high throughput, multiparametric technique that combines the speed and sensitivity of conventional MFC with visual information from fluorescence microscopy. The ImageStream^®X^ MKII (ISX, Cytek Biosciences, Fremont, CA, USA) instrument captures 12 high-resolution images per cell, including two brightfield (BF) images, one darkfield/SSC image, and nine fluorescence images. As such, IFC permits analysis of image data, enabling investigation of morphometric characteristics of hundreds of thousands of cells per sample. For each cell, thousands of features, such as size, shape, and texture, can be calculated [34,35,36]. When combined with AI, the statistical sensitivity of the technique is greatly improved [37,38,39]. This high-content analysis makes IFC an ideal methodology for evaluating rare cellular subsets. In the current study, we applied IFC in combination with convolutional neural network (CNN)-based image analysis to morphologically discriminate CLEC12A^+^ LSCs and healthy CLEC12A^−^ HSCs. We show proof-of-concept results of robust AI-based HSC-LSC differentiation, helping pave the way for the establishment of novel techniques within AML monitoring.

## 2. Results

### 2.1. IFC Permits Immunophenotypic Identification of HSCs and LSCs

**Figure 1:**
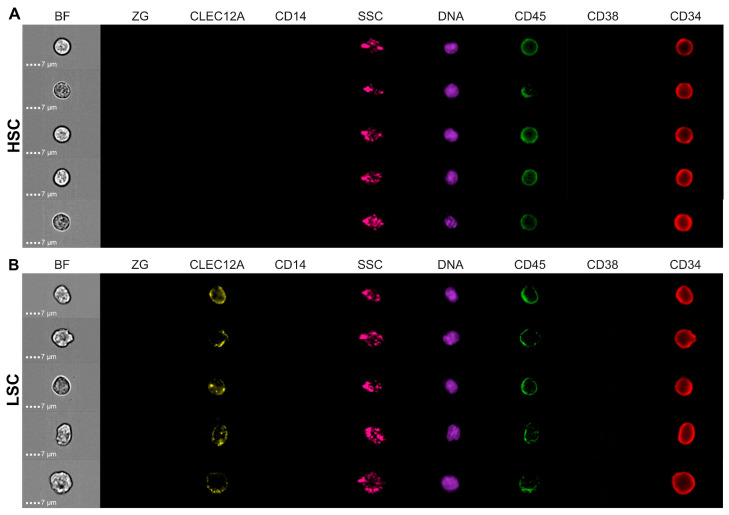
Immunophenotypically detected HSCs and LSCs. A seven-color IFC panel was designed to enable detection of HSCs and LSCs in BM samples. Five AML samples and ten NBM samples were stained with all pre-titrated antibodies, acquired on the ISX, and analyzed in IDEAS software (V6.3.41.0). A series of gating steps was performed, employing the immunophenotypic profiles of HSCs and LSCs. Stem cells were identified as CD34^+^CD38^−^CD45^low^CD14^−^ live, nucleated, single cells in focus. Within NBM samples, this population constituted HSCs, while LSCs in AML samples were further identified as CLEC12A^+^. Representative examples of images recorded in all utilized channels on the ISX are shown of (**A**) HSCs from NBM samples and (**B**) LSCs from AML samples. Every row represents one cell, while each column shows either BF, SSC, or fluorescence images of a specific marker. Abbreviations: AML; acute myeloid leukemia, BF; brightfield, BM; bone marrow, HSC; hematopoietic stem cell, IFC; imaging flow cytometry, ISX; ImageStream^®X^ MKII, LSC; leukemic stem cell, NBM; normal bone marrow, SSC; side scatter.

A seven-color IFC panel was designed and utilized to identify HSCs and LSCs in BM samples from ten healthy donors and five AML patients, respectively. The immunophenotypic profiles of these cell types were employed to perform a series of gating steps leading to the populations of interest. Initially, stem cells were identified as CD34^+^CD38^−^CD45^low^CD14^−^ among live, nucleated, single cells in focus. While these constituted healthy HSCs in normal BM (NBM) samples (Figure 1A), a subset of LSCs were further identified in AML samples as showing aberrant expression of the marker CLEC12A (Figure 1B). Thus, we demonstrated the feasibility of a seven-color HSC-LSC tube to immunophenotypically detect HSCs and CLEC12A^+^ LSCs using IFC.

### 2.2. AI-Based Image Analysis Enables Accurate HSC-LSC Discrimination

**Figure 2:**
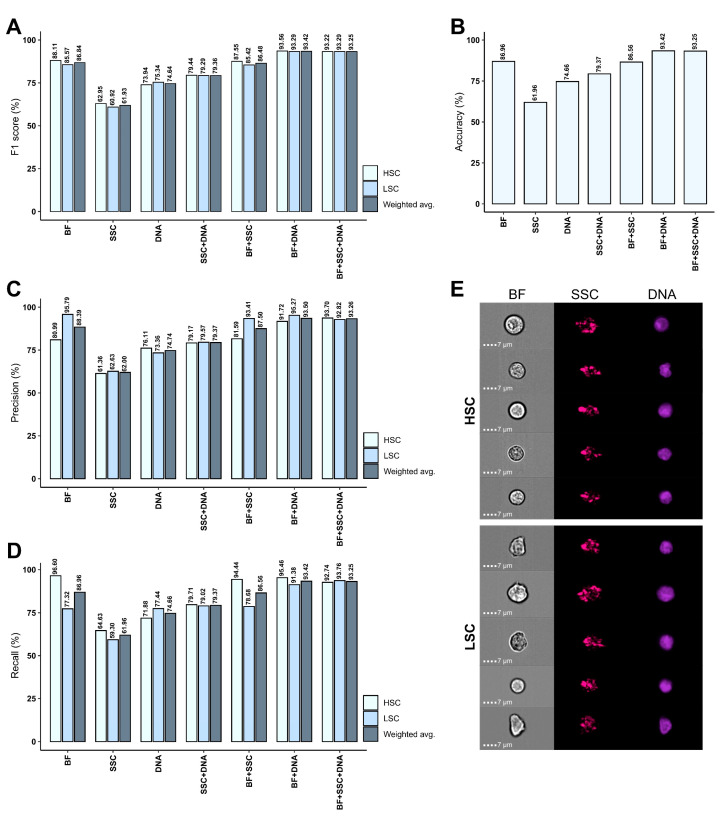
Evaluation metrics for classification of test data. Seven image classifiers were trained to distinguish between healthy HSCs and aberrant LSCs from NBM and AML samples, respectively, based on various combinations of image data: BF, SSC, DNA, SSC + DNA, BF + SSC, BF + DNA, or BF + SSC + DNA. A CNN model architecture and the train-validation-test split technique were applied. The models were evaluated using results from classification of the test data that was not previously seen by the models during training (*n* = 1764). Presented are (**A**) F1 scores, a harmonic average of precision and recall (i.e., (2·precision·recall)/(precision + recall)), (**B**) accuracy, calculated for all models as the percentage of correct predictions (i.e., (TP + TN)/(TP + TN + FP + FN)), (**C**) precision, defined as the proportion of correctly classified events (i.e., TP/(TP + FP)), (**D**) recall, also known as the sensitivity/true positive rate (i.e., TP/(TP + FN))**.** All metrics are shown for HSCs and LSCs separately and as a weighted average. (**E**) Example images show true HSCs (**top rows**) and true LSCs (**bottom rows**). Each row corresponds to one cell and columns show BF (**left**), SSC (**middle**), and DNA images (**right**) captured using the ISX in Ch01, Ch06, and Ch07, respectively. Abbreviations: AML; acute myeloid leukemia, BF; brightfield, Ch; channel, CNN; convolutional neural network; FN; false negative, FP; false positive, HSC; hematopoietic stem cell, ISX; ImageStream^®X^ MKII, LSC; leukemic stem cell, NBM; normal bone marrow, SSC; side scatter, TN; true negative, TP; true positive.

**Figure 3:**
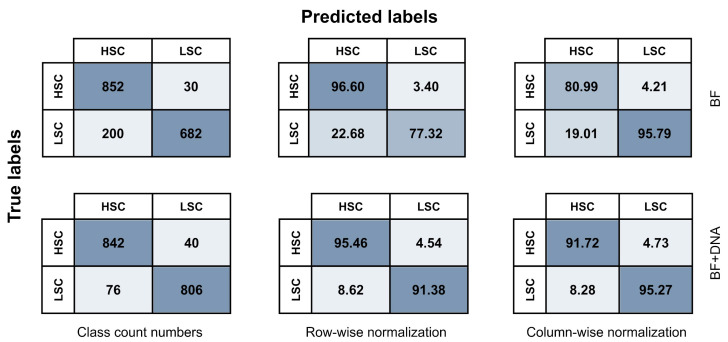
Confusion matrices. All stem cells in the test data (*n* = 1764) were classified as HSCs or LSCs by the BF model (**top**) and the BF + DNA model (**bottom**). The relationship between true and predicted labels are shown as class count numbers, specifying TP, TN, FP, and FN events (**left**). Row-wise normalization was conducted, delineating recall/sensitivity for each category (**middle**). Colum-wise normalization was applied, showing precision for each category (**right**). Corresponding confusion matrices can be found for all seven AI models in Figure S1. Abbreviations: BF; brightfield, FN; false negative, FP; false positive, HSC; hematopoietic stem cell, LSC; leukemic stem cell, SSC; side scatter, TN; true negative, TP; true positive.

Considering the prognostic value of LSCs and their substantial immunophenotypic heterogeneity both within and between AML samples [14,15], novel methods are required to accurately identify and discriminate LSCs from their healthy HSC counterparts. As an exploratory strategy, we set out to differentiate between these two cell types using AI-driven image analysis. We successfully developed AI models using a CNN algorithm to classify stem cells into either of the following two categories: (i) healthy HSCs or (ii) aberrant LSCs. Seven different models were trained for HSC-LSC discrimination using various combinations of BF, SSC, and DNA image data from immunophenotypically defined HSC and LSC populations. Evaluation metrics including F1 scores, accuracy, precision, and recall were obtained for both training, validation, and test sets resulting from splitting the total data by an 80/10/10 ratio (Table 1 and Figure 2). Moreover, the relationship between true and predicted labels were recorded in confusion matrices for the test data (Figure 3 and Figure S1).

Testing of the model created solely using BF images showed an overall F1 score of 86.84% (Table 1 and Figure 2A) and accuracy of 86.96% (Figure 2B), revealing considerable AI-detected morphologic differences between HSCs and LSCs. Of all LSC and HSC predictions, 95.79% and 80.99%, respectively, were correct (Table 1, Figure 2C and Figure 3). It was possible to correctly retrieve 96.60% of all HSCs and 77.32% of all LSCs, showing a higher recall/sensitivity for HSCs (Table 1, Figure 2D and Figure 3). This was attributable to misclassification of 22.68% of LSCs as HSCs, while only 3.40% of HSCs were misclassified as LSCs (Figure 3). Thus, the BF model primarily showed high HSC recall and high LSC precision. Next, we assessed the model created using only DNA images and observed that the evaluation metrics ranged between 71.88% and 77.44% with an overall F1 score of 74.64% and accuracy of 74.66% (Table 1 and Figure 2A–D). While these results indeed designate some differences in nuclear morphology between the two classes, it also demonstrates that the DNA images alone are insufficient for accurate HSC-LSC separation due to misclassification of 28.12% of HSCs and 22.56% of LSCs (Figure S1). Model development solely using SSC images revealed only subtle dissimilarities in granularity between HSCs and LSCs with an overall F1 score of 61.93%, accuracy of 61.96%, and precision and recall ranging between 59.30% and 64.63% (Table 1, Figure 2A–D and Figure S1).

When training was based on a combination of BF and DNA images, evaluation metrics ranged from 91.38% to 95.46%, signifying notably high performance statistics for both classes (Table 1 and Figure 2A–D). The F1 score and accuracy based on the test data were both 93.42% (Table 1 and Figure 2A,B). Moreover, incorporating DNA images alongside BF images resulted in better classification balance between the two classes compared with using BF images alone. Still, precision was slightly higher for LSCs (Table 1, Figure 2C and Figure 3) and recall was slightly higher for HSCs (Table 1, Figure 2D and Figure 3). Only 4.54% of HSCs and 8.62% of LSCs were misclassified (Figure 3). Thus, the BF + DNA model offered superior performance. Of note, inclusion of SSC images into model training offered either no or only minor improvements in performance when compared to utilizing the other images without SSC. This was the case both when combined with BF images and DNA images individually or together (Table 1, Figure 2A–D and Figure S1).

### 2.3. Model Performance Varies Substantially between AML Patients

Thus far, all presented model performance metrics were based on pooled HSC and LSC data sets from multiple NBM and AML samples, respectively. However, an important question remains—whether any noteworthy morphologic differences exist between CLEC12A^+^ LSCs identified from different AML patients and whether this may cause variation in patient-specific image classification accuracy. Hence, we investigated any differences in model performance by assessing evaluation metrics separately for four AML patients. An additional 8487 CLEC12A^+^ LSCs that had not been included in previous analyses were classified as either LSCs or HSCs using all seven developed AI models, and class predictions were recorded both for the AML patients separately and combined.

**Figure 4:**
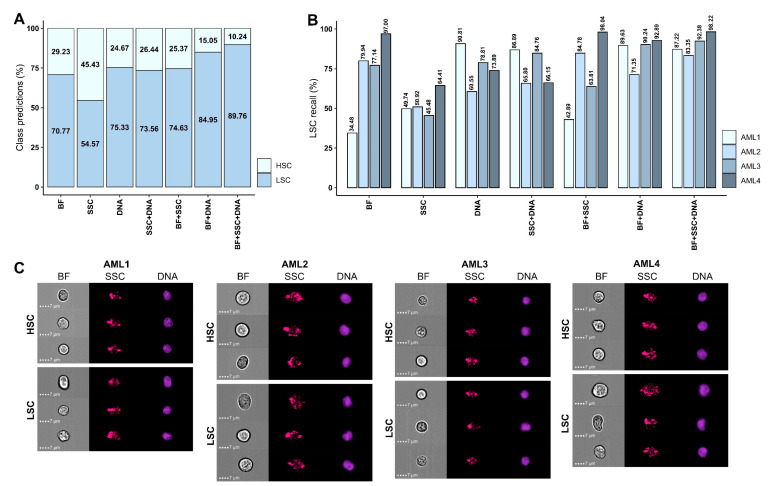
Patient-specific classification of LSCs. All seven AI models developed for HSC-LSC differentiation were evaluated further by classification of 8487 immunophenotypically defined CLEC12A^+^ LSCs identified in BM samples from four AML patients (AML1 *n* = 2700, AML2 *n* = 2667, AML3 *n* = 420, AML4 *n* = 2700). These were not previously included in the data sets used for training, validation, or testing. All CLEC12A^+^ LSCs were classified based on appropriate combinations of BF, SSC, and DNA images. (**A**) Class predictions are shown as stacked bar graphs designating the percentages of stem cells classified as either LSCs or HSCs by each model. (**B**) The results were evaluated on a per-patient basis, and the percentage of cells classified as LSCs, corresponding to LSC recall, are indicated for each AML patient. (**C**) Examples are shown of stem cells classified as HSCs (**top rows**) or LSCs (**bottom rows**) by the BF + SSC + DNA model for each of the four AML patients. Each row corresponds to one cell and columns show BF (**left**), SSC (**middle**), and DNA images (**right**) captured using the ISX in Ch01, Ch06, and Ch07, respectively. Abbreviations: AI; artificial intelligence, AML; acute myeloid leukemia, BF; brightfield, BM; bone marrow, Ch; channel, HSC; hematopoietic stem cell, ISX; ImageStream^®X^ MKII, LSC; leukemic stem cell, SSC; side scatter.

The percentage of LSCs identified among all AML patients combined, corresponding to LSC recall, ranged between 54.57% and 89.76%. This was attributable to the misclassification of 10.24–45.43% of LSC as HSCs (Figure 4A). Based on BF images, it was possible to correctly identify 70.77% of LSCs, while using DNA images alone retrieved 75.33% of LSCs. The addition of DNA images to BF images increased LSC recall to 84.95%, and a slightly higher predicted LSC percentage of 89.76% resulted from the assessment of a combination of BF, SSC, and DNA images. Classification based on SSC images alone, however, neither enabled accurate LSC identification nor improved the BF model notably.

Assessment of performance metrics for each AML patient separately revealed noticeable differences (Figure 4B), pointing towards prominent morphologic variation between CLEC12A^+^ LSCs identified from different AML patients. Interestingly, BF image-based classification enabled identification of only 34.48% of LCSs from AML1 but nearly all LSC from AML4 with an LSC recall of 97.00%. For AML2 and AML3, the metrics were similar, with 79.94% and 77.14% LSCs correctly classified, respectively, based on BF images. Conversely, using DNA images, 90.81% of LSCs from AML1 were identified, while this ranged from 60.55% to 78.81% for the three other AML patients. Combining BF and DNA images resulted in very high percentages of predicted LSCs, between 89.63% and 92.89% for AML patients 1, 3, and 4, although this remained lower for AML2, with 71.35% LSCs identified. SSC images were generally insufficient for correct classification, as only 45.48–64.41% LSCs were recalled. Combining SSC with BF or DNA images either slightly increased or slightly decreased LSC recall compared with using BF or DNA images alone. Nonetheless, using a combination of all three images, the BF + SSC + DNA model was able to correctly classify between 83.35% and 98.22% of LSCs, showing higher balance and robustness across patient samples, contrasting all other models.

## 3. Discussion

In this exploratory study, we investigated the ability of AI-driven image classification to distinguish between CLEC12A^+^ LSCs from AML BM and HSCs from healthy controls using BF, SSC, and DNA imagery. We present proof-of-concept data showing that CLEC12A^+^ LSCs and healthy HSCs could be discriminated with accuracy, precision, and recall metrics of approximately 93% using a CNN classifier based on BF, SSC, and DNA images (Table 1 and Figure 2). While combining all three image types resulted in the highest performance metrics, discrimination based on BF images alone also offered a high accuracy of 86.96% (Table 1 and Figure 2). Of all LSCs, 95.79% were correctly classified, and 96.60% of HSCs could be recalled using the BF model (Figure 2 and Figure 3). Collectively, these results highlight notable morphological differences between LSCs and HSCs. This aligns with prior MFC analyses, which indicated increased FSC/SSC properties in AML LSCs relative to HSCs, suggesting variations in refractive index, internal complexity, size, and granularity inherent to morphological properties, all of which are critical elements influencing FSC/SSC signal intensities [4,22,40]. However, although Terwijn et al. reported distinctions based on SSC properties [4,22], our study found that this parameter alone enabled far inferior HSC-LSC discrimination compared to BF and DNA images. While SSC images alone allowed for the accurate classification of 61.96% of stem cells, combining SSC and BF images did not improve results beyond those obtained with BF images alone (Table 1 and Figure 2). Discrepancies between studies may be due to differences between MFC-generated SSC signals and IFC-based SSC imagery. Using DNA images alone enabled accurate designation of 74.66% of stem cells, while incorporating DNA imagery into classification based on BF images improved model accuracy to 93.42% (Table 1 and Figure 2). This indicates that noticeable nuclear differences exist between HSCs and LSCs. We applied the VDCV dye, which stains nuclei by binding to double-stranded DNA. Variances in VDCV intensities were observed across the BM samples included in our study, with AML patient samples exhibiting stronger signals compared to healthy donor samples. Consequently, compensation of spillover signals from this dye into other channels was required on a patient-specific basis. Speculatively, these differences may stem from inherent variations in DNA, including accessibility, as transformation of HSCs into LSCs is caused by chromosomal alterations, mutations, or changes in epigenetics [41]. Also, it has been demonstrated that HSCs can efflux the VDCV dye via a membrane pump-dependent mechanism, giving rise to a side population (SP) with reduced fluorescence intensity, while the dye tends to be retained within more differentiated cells [42]. Telford et al. previously utilized this phenomenon for identification of stem cells and labeled it the SP technique [42]. Although CD34^+^ AML contains LSCs within a CD34^+^CD38^−^ SP [43], it remains unknown whether differences in the extent of dye efflux might have contributed to the observed nucleic differences between HSCs and LSCs in our study.

AML is a heterogeneous disease characterized by various molecular traits, immunophenotypic profiles, and variations in the cell-of-origin and maturation stage [6,13]. These differences may be reflected in LSCs as morphological diversity. Given this complexity, we selected AML patients with similar disease characteristics by the inclusion of only CD34^+^ and de novo cases. However, there is considerable cytogenetic and molecular heterogeneity between patient samples, reflecting the actual diversity of AML. To accommodate this, LSCs from each AML case were included in equal numbers during model training. Nevertheless, investigating LSC classification results for four AML patients separately revealed marked differences in model performance. For example, LSC recall ranged between 34.48% and 97.00% when classifying solely based on BF images (Figure 4). Hence, our results indicate that CLEC12A^+^ LSCs from different AML patients show morphological differences. This raises the question of how many distinct AML cases should be included to adequately capture the inter- and intra-patient variations, which could impact the accuracy of the developed classifier. Thus, even if it is possible to discriminate LSCs and healthy HSCs by our methodology, it may not work for other AML types that harbor LSCs with different morphological characteristics. Healthy HSCs, on the other hand, are presumably morphologically similar, although this is yet to be investigated. It may therefore be preferable to employ AI-based recognition of HSCs rather than LSCs when searching for residual disease within AML BM, in which case LSCs would be defined as non-HSCs. This could possibly result in a higher balance in performance metrics across patient samples and could obviate the need for patient-specific assays. Importantly, as HSCs were derived from healthy donor BM samples, our strategy assumes that these are morphologically similar to healthy HSCs co-residing with aberrant LSCs within the stem cell compartment of AML samples. However, it is possible that HSCs and LSCs may exhibit inherently similar morphology within the same patient, despite the malignant transformation leading to LSC formation. If this is the case, the HSC-LSC discrimination strategy proposed in this manuscript could be prone to misclassification, at least in some patient samples. While it would be optimal to instead compare LSCs to HSCs from AML BM, such a strategy remains unfeasible in the current study, as the absence of an LSC-marker does not necessarily equal the absence of malignancy [31]. Identifying HSCs within AML BM would require cell sorting followed by molecular and genetic analyses as well as functional tests, but this is currently not a possibility when using IFC. Also, within AML BM, the CD34^+^CD38^−^ stem cell compartment may harbor blasts alongside HSCs and LSCs, as blasts may display marker positivity corresponding to their cell-of-origin. In the current study, LSCs were identified by CLEC12A positivity, although CLEC12A is often expressed on leukemic blasts at the time of AML diagnosis [44]. Ensuring optimal immunophenotypic delineation of blasts requires an extensive panel, including markers of monocytic (e.g., CD11b, CD64, CD4), erythroid (e.g., CD71, CD105, CD117), and megakaryocytic differentiation (e.g., CD61, CD42b) [45]. Alternatively, it may be possible to train an AI model to detect blasts using morphology. This has indeed been attempted by Matek et al., who trained a CNN model to recognize myeloblasts in peripheral blood smears from AML patients with precision and sensitivity metrics of 94% [46]. However, accurate distinction between blasts and LSCs ultimately requires functional assays [5,27]. 

The truth populations for CNN model training in the current study were created by the immunophenotypic detection of HSCs and LSCs within samples from healthy donors and AML patients, respectively (Figure 1). HSCs were identified by gating of the CD34^+^CD38^−^ stem cell compartment in NBM. In general, CD34 identifies BM cells that are capable of reconstituting the entire hematopoietic system, which is currently exploited successfully in hematopoietic stem cell transplantation [47,48]. Enrichment of HSCs improves further by selecting CD38^−^ cells, as CD34^+^CD38^−^, but not CD34^+^CD38^+^ BM cells, are capable of long-term production of both myeloid and lymphoid cells [48,49,50,51,52,53]. While both HSCs and their immediate downstream progeny (i.e., multipotent progenitors) are CD34^+^CD38^−^, further differentiation stages (i.e., common lymphoid progenitors, common myeloid progenitors, megakaryocyte-erythroid progenitors, and granulocyte-macrophage progenitors) are excluded by being CD34^+^CD38^+^ [54]. Although results are conflicting, various studies also suggest the existence of some HSCs within the CD34^−^ fraction [48]. LSCs co-reside with HSCs within the CD34^+^CD38^−^ compartment. While LSCs are enriched within the aberrant marker-positive CD34^+^CD38^−^ population [21,29,55,56,57], leukemia initiating cells can be found within all CD34/CD38 combinations in AML [57,58,59,60,61,62]. However, it is not possible to immunophenotypically discriminate LSCs from healthy progenitor cells within the CD34^+^CD38^+^ compartment, as the latter express several markers that are also aberrantly expressed on LSCs (e.g., CD45RA, CD123, and CLEC12A) [32]. We identified LSCs by selecting CD34^+^CD38^−^ stem cells expressing the aberrant marker CLEC12A. Previously, the presence of CLEC12A on LSCs has been reported in 92% of AML cases, while it is absent on healthy CD34^+^CD38^−^ HSCs and during BM regeneration [25,44]. However, multiple other LSC markers have been described, including CD123, CD7, and CD19 [15,23,63]. Due to the considerable heterogeneity between and within AML samples [30], we refrained from following a one-tube approach as performed by Zeiljemaker et al. [22,63], where multiple LSC markers are placed in a single channel. While some LSC markers have a clear boundary between positive and negative populations (e.g., CD7, CLEC12A, and CD45RA), others may show continuous expression levels and/or overexpression compared with healthy cells (e.g., CD33, CD47, and CD96) [5]. Additionally, the lack of specific aberrant markers is not necessarily equal to the absence of malignant characteristics [5]. Based on these considerations, we decided to include a single well-established LSC marker, namely CLEC12A. However, it is unknown whether CLEC12A^+^ LSCs are morphologically similar to LSCs displaying positivity of other aberrant markers, which may impact the feasibility of model extrapolation. If our models are applied to classify stem cells within the entire CD34^+^CD38^−^ stem cell compartment of AML samples, it is likely that multiple other LSCs are identified that were not originally detectable by expression of CLEC12A. An important question in this regard remains how to validate the correct designation of LSCs. This would require isolation of LSCs based on BF, SSC, and/or DNA images followed by molecular and cytogenetic examinations of the presence or absence of leukemia-defining aberrations. In addition, functional tests are needed to prove LSC status irrevocably. Label-free AI-powered systems for cell sorting based on morphology, e.g., ThinkCyte and Deepcell, have recently emerged and are intriguing possibilities for LSC isolation. These novel systems offer unique capabilities of combining single cell imaging with label-free classification and sorting using computer vision [64,65].

Given the prognostic value of LSCs [4], reliable methods for HSC-LSC discrimination are highly sought after. Especially in a clinical setting, maintaining a high sensitivity and specificity is of utmost importance. Both diagnostic procedures and MRD monitoring require robust assays, particularly as some follow-up BM samples encompass only few CD34^+^CD38^−^ cells [22]. This underlines the need for decreasing FN and FP results when discriminating between HSCs and LSCs. As the frequency of neoplastic CD34^+^CD38^−^ stem cells holds prognostic impact [4], misclassification of HSCs as LSCs may falsely indicate disease initiation or recurrence. Oppositely, misclassification of LSCs as HSCs may falsely point towards absence of malignant disease. In our study, the BF model showed differing FN and FP results when used for LSC detection, with as many as 22.68% of LSCs misclassified compared with only 3.40% of HSCs (Figure 4). By using a combination of BF, SSC, and DNA images to detect LSCs, it was possible to decrease the FP rate to 6.24%, although at the expense of increasing the FN rate to 7.26% (Figure S6). This poses the consideration of whether future optimization of the model should focus on decreasing misclassification of LSCs or HSCs. One option could be lowering the prediction threshold for assignment of stem cells as LSCs, which may increase clinical relevance by lowering the FN rate in this regard. Given the acute nature of AML, it could be argued that an emphasis should be placed on increasing sensitivity, as not to falsely dismiss return of aggressive malignancy [66]. Also, only discrimination between HSCs and LSCs has been discussed, while the number of detected LSCs required to define a patient as AML/MRD positive needs to be determined. The potential applicability for MRD measurements and prognostics should be examined by detection of LSCs in follow-up BM and correlation with clinicopathological features and outcomes, including relapse. The technique may provide an alternative or an addition to other MRD methods, such as MFC and RT-qPCR.

From a biological viewpoint, it would be of great interest to elucidate which cellular features our results are attributable to. The structure of a neural network is essentially a black box that cannot be easily understood [67]. Therefore, our current work does not enable interpretation of which features HSC-LSC distinction relies upon. Testing of AI models with higher interpretability is a sensible next step, and we expect that it will shed further light on the specific dysmorphological characteristics of LSCs. Moreover, experimenting with crucial characteristics of the CNN architecture, e.g., filters, weights, and the number of neurons, may enable improvements in model performance. Classification may also be enhanced by expanding the ground truth data provided for training. Prior to model training, we performed class balancing by under-sampling, meaning that we discarded data from the abundant class. This was performed to avoid bias towards the majority class of LSCs. However, it resulted in utilization of only 8818 out of 44,277 available LSCs. Although these were randomly sampled in almost equal numbers from each AML patient, presumably conserving the nuances in LSC morphology, this strategy resulted in far less extensive ground truth data. Different options include augmentation of minority class data, over-sampling by duplicating minority class data, or employing cost-sensitive training where minority class mistakes are penalized proportionally to how underrepresented it is. Also, introducing a class representing stem cells that cannot be confidently classified as neither LSCs nor HSCs may have been highly beneficial, which would require setting a classification threshold. However, these techniques for improving model classification are currently not possible within the Amnis AI (AAI) software (V2.1.30) used in this study, as it is designed primarily for simplicity, enabling individuals without prior knowledge of AI and coding to employ deep learning [68]. Alternative software options include CellProfiler combined with TensorFlow and Keras [35,37,39,69,70]; DeepFlow [71]; and Deepometry [72].

Collectively, IFC holds unique potential for combining immunophenotyping and morphological investigation. Multiple other studies have applied this technique to investigate label-free approaches for analysis of blood cell types. In a study by Doan et al., the capability of using IFC combined with deep learning has been demonstrated by identifying leukemic cells in BM samples from children with acute lymphocytic leukemia with an accuracy of 88% solely using BF- and SSC-based features [39]. An AI-assisted IFC method has been developed by Nassar et al. for feature-based classification of white blood cells into eosinophils, lymphocytes, monocytes, and neutrophils with an F1 score of 97%. T and B cells could be further discriminated with an F1 score of 78% [37]. Masking of BF images has allowed proper distinguishment of leukocyte-platelet aggregates from coincidental events [73], and the technique has been further exploited to assess the quality of erythrocytes for blood transfusions [74]. Specifically, within our research group, Rosenberg et al. demonstrated the applicability of using IFC to characterize dyserythropoiesis by analysis of morphometrics within myelodysplastic syndrome samples. Rare binucleated erythroblasts were identified by combining IFC with either feature-based machine learning or a CNN, showing comparable quantification accuracy [75,76]. In our study, we combined IFC and CNN-based image analysis to morphologically discriminate aberrant LSCs and healthy HSCs with an accuracy of 93%. Thus, we have provided proof-of-concept results of a novel AI-based HSC-LSC differentiation technique. Although promising and paving the way for further investigations, additional data and optimization are needed before it can be considered for monitoring of AML patients and possibly even other hematological malignancies. In many cases, rather than being a stand-alone-technique, IFC-based image analysis may prove an important addition to other methodologies.

## 4. Materials and Methods

### 4.1. Bone Marrow Samples

All LSCs and HSCs were obtained from BM samples from either AML patients or healthy donors, respectively. Diagnostic cryopreserved BM samples from a total of five AML patients were included in the study (Table 2). Inclusion criteria for AML patients were de novo AML, CD34 positivity, and ≥0.025% CLEC12A^+^ LSCs out of the total number of events acquired during routine flow cytometric analysis (Figure S2). All AML samples were excess material taken as part of the diagnostic process at the Department of Hematology, Aarhus University Hospital (AUH), Denmark. In addition, cryopreserved BM samples from ten healthy donors (NBM) were included in the study. These samples were either excess material collected during BM harvesting for allogenic stem cell transplantation or were donated to “The Anonymous Biobank for Normal Donors at the Department of Hematology”. The study was approved by the Danish Data Protection Agency (record no.: 1-16-02-849-17) and the Central Denmark Region Committee on Health Research Ethics (record no.: 1-10-72-125-17) and performed in accordance with the Declaration of Helsinki.

### 4.2. Sample Preparation

Mononuclear cells (MNCs) were harvested from BM aspirates by density gradient centrifugation on Lymphoprep (Alere Technologies AS, Oslo, Norway). Erythrocytes were lysed using lysis buffer (Ampliqon, Odense, Denmark). This was followed by cryopreservation in a storage solution containing 10% DMSO (Sigma-Aldrich/Merck, St. Louis, MO, USA), 50% fetal calf serum (FCS) (Biowest, Nuaillé, France), and 40% RPMI (Invitrogen, Waltham, MA, USA), then stored in liquid nitrogen tanks at −165 °C. Ampoules were thawed by partial submersion in water at 37 °C for one minute. Cells were washed and resuspended in stain buffer (SB) containing 2% FCS (Biowest) and 98% Hank’s balanced salt solution (HBSS) (ThermoFisher, Waltham, MA, USA). Then, MgCl_2_ (Sigma-Aldrich) and DNase (Sigma-Aldrich) were added to final concentrations of 5 mM and 100 Kunitz units/mL, respectively. The samples were incubated for 10 min at room temperature (RT) or at 37 °C if containing clumps. If clumps consisted, the solution was filtered (Miltenyi Biotec, Bergisch Gladbach, Germany). Cell counting was performed using a Sysmex XP-300^TM^ Automated Hematology Analyzer (Sysmex, Kobe, Japan). The volume of the remaining cell suspension was measured, and SB was added to obtain a final concentration of 80·10^6^ white blood cells/mL. Fc-blocking was performed using Privigen (CSL Behring, Kongens Lyngby, Denmark), added in a final concentration of 100 µg/mL followed by 10 min of incubation at 4 °C [77].

### 4.3. Staining Procedure

A seven-color IFC panel was designed for identification of HSCs and LSCs in BM samples. Thawed BM MNCs were stained with the following pre-titrated monoclonal antibodies: CLEC12A PE (Clone HB3, created in-house and conjugated by Agilent, Santa Clara, CA, USA), CD14 PE-Texas Red (Clone Tuk4, ThermoFisher), CD45 KrO (Clone J33, Beckman Coulter, Brea, CA, USA), CD38 SBV610 (Clone AT13/5, Bio-Rad, Hercules, CA, USA), CD34 AF647 (Clone 581, BioLegend, San Diego, CA, USA), and Zombie Green (ZG) Viability Dye (BioLegend) (Table S1). Samples were incubated for 30 min at 4 °C in the dark and washed twice. This was followed by staining of DNA with VDCV (Invitrogen) and incubation for 30 min at 37 °C without subsequent washing. Additionally, three control samples were created for each AML and NBM sample: a fluorescence minus two (FM2) sample stained with all antibodies and dyes except CD38 SBV610 and CLEC12A PE, a sample stained solely with VDCV, and an unstained control.

### 4.4. IFC Configuration and Acquisition

All samples were acquired on the ISX (Cytek Biosciences) at the FACS Core Facility, Aarhus University (AU), Denmark. A data acquisition template was created in the INSPIRE^®^ software (V200.1.620.0, Cytek Biosciences). The following laser power settings were selected: 405 nm; 120 mW, 488 nm; 200 mW, 642 nm; 150 mW, and 785 nm (SSC); 1 mW. BF images were captured in Ch01 and Ch09 and SSC signal in Ch06. Each stain was detected as follows: ZG in Ch02, CLEC12A PE in Ch03, CD14 PE-Texas Red in Ch04, VDCV in Ch07, CD45 KrO in Ch08, CD38 SBV610 in Ch10, and CD34 AF647 in Ch11. Consecutive raw image files (.rif) were obtained at low speed and a magnification of 60x. The number of replicas per BM sample depended on the amount of available sample material. A median of 7 (range: 7–21) and 14.5 (range: 8–19) replicas were collected for AML and NBM samples, respectively. Additionally, 20,000 singlets were collected from each unstained control sample, 50,000 singlets from each FM2, and 20,000 singlets from each sample stained solely with VDCV. As samples were acquired on different days, instrument performance was validated prior to each run using Rainbow Calibration Particles (Spherotech, Lake Forest, IL, USA). The median fluorescence intensity of peak six was measured and compared with a baseline interval. Single-stained compensation controls were created using Quantum Simply Cellular Beads (anti-mouse IgG, Bangs Laboratories Inc, Fishers, IN, USA) for all antibodies and MNCs for viability and DNA dyes. Sample-specific VDCV compensation controls were created due to varying DNA signals. The INSPIRE^®^ compensation wizard was utilized to collect 2000 positive singlets from each sample, followed by creation of a compensation matrix in the IDEAS^®^ software (V6.3.41.0, Cytek Biosciences) using the best fit linear regression method. A compensation matrix was subsequently applied to each acquired .rif file prior to analysis.

### 4.5. Gating of Healthy and Leukemic Stem Cell Populations

**Figure 5:**
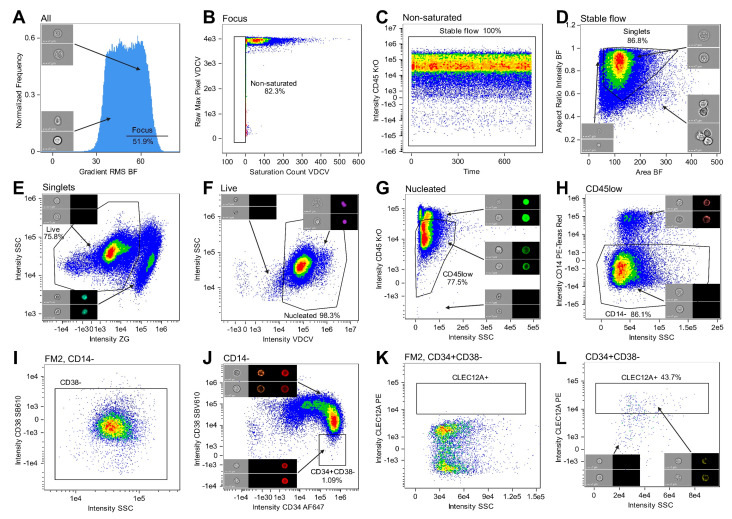
Gating strategy. The series of gating steps leading to identification of stem cells and CLEC12A^+^ LSCs is shown for a representative AML sample. (**A**) Using gradient RMS values of BF images along with visual inspection of images, cells in focus were selected. (**B**) Images without saturated pixels were gated as events having a raw max pixel feature below 4096 and/or a saturation count below one in each channel. Specifically shown in this example is Ch07. (**C**) Any events in unstable flow were excluded based on the distribution of cells in a plot showing the time parameter. (**D**) Singlets were gated based on the features area and aspect ratio, excluding aggregates and debris. (**E**) Live cells were identified as showing negative or low ZG signals. (**F**) We selected nucleated cells as VDCV^+^. (**G**) In a bivariate plot showing intensity SSC and intensity CD45 KrO, a population of CD45^low^ cells including blasts, HSCs, and LSCs was selected by exclusion of CD45^−^ events, CD45^high^SSC^low^ lymphocytes, and any residual SSC^high^ granulocytes. (**H**) Exclusion of monocytes was conducted by gating CD14^−^ cells. (**I**) The threshold specifying the distinction between CD38^+^ and CD38^−^ events was set based on the FM2. (**J**) Finally, CD34^+^CD38^−^ stem cells were selected. (**K**) For AML samples, a CLEC12A^+^ gate was created based on the FM2. (**L**) Subsequently, the CLEC12A boundary was applied to the stem cells, thereby identifying CLEC12A^+^ LSCs. The percentage gated of the input population is indicated in each plot. Abbreviations: AML; acute myeloid leukemia, BF; brightfield, FM2; fluorescence minus two, HSC; hematopoietic stem cell, IFC; imaging flow cytometry, LSC; leukemic stem cell, RMS; root mean square, SSC; side scatter, ZG; zombie green, VDCV; vybrant dyecycle violet.

Based on the fact that CLEC12A is not expressed on healthy HSCs [32], we defined CLEC12A^+^CD34^+^CD38^−^ cells from AML samples as LSCs and CLEC12A^−^CD34^+^CD38^−^ cells from healthy BM as HSCs. Gating of all samples was performed using the IDEAS^®^ software (V6.3.41.0, Cytek Biosciences). Initially, cells in focus were selected based on gradient root mean square (RMS) values of BF images. Gating was guided by visual inspection of BF images in each histogram bin (Figure 5A). Then, the files were checked for images that included saturated pixels. For each fluorescence channel, we gated events having a raw max pixel feature value below 4096 and/or a saturation count below one (Figure 5B). Any cells in unstable flow were excluded using the time parameter (Figure 5C). A single cell gate was created using the area and aspect ratio, by which cellular aggregates and debris were excluded (Figure 5D). Live cells were gated as ZG^neg–dim^ (Figure 5E) and nucleated cells as VDCV^+^ (Figure 5F). Next, CD45^low^CD14^−^CD34^+^CD38^−^ stem cells were identified. A gate was placed around the CD45^low^ population in a bivariate plot showing SSC signals against CD45 KrO intensity (Figure 5G). The CD45 KrO intensity feature was based on a mask customized to select pixels corresponding to the cell membrane (Figure S3). Any monocytes remaining in the population were excluded by gating CD14^−^ cells (Figure 5H). Finally, the CD34^+^CD38^−^ stem cell compartment was selected (Figure 5J), from which a subset of LSCs were identified as CLEC12A^+^ in AML samples (Figure 5L). The FM2 was used to define the boundary for positivity of CD38 SBV610 and CLEC12A PE (Figure 5I,K). Lastly, any clipped images or images that contained debris fragments along with the cell were excluded as those with a circularity feature ≤ 5 (Figure S4). All masks and features are described in detail in Figure S5 and Table S2.

### 4.6. Data Partitioning and Class Balancing

A total of 44,277 CLEC12A^+^ LSCs and 8818 CLEC12A^−^ HSCs were available from five AML and ten NBM samples, respectively. To avoid bias towards the majority class of LSCs, class balancing was performed manually prior to model training by reducing the LSC sample size to 8818 randomly selected LSCs with approximately equal representation among the individual AML samples. Thus, the final dataset consisted of 8818 CLEC12A^+^ LSCs and 8818 CLEC12A^−^HSCs (Table 3).

### 4.7. CNN Model Development Using Amnis^®^ AI

Deep learning was performed using the AAI software (V2.1.30, Cytek Biosciences). This applies the Keras Application Programming Interface (V2.1.5) with TensorFlow (V1.7.0) and uses a CNN model architecture based on the VGG16 network [78,79,80]. The process of creating ground truth populations and training new CNN models within the AAI software has been described in detail in several recent publications [68,76,78]. In the current study, we applied this software to train a CNN to distinguish between CLEC12A^+^ LSCs and CLEC12A^−^ HSCs based on images captured using IFC. Parameters included were Ch01 BF images, Ch06 SSC images, and Ch07 DNA images. Seven different CNN models were created based on the following combinations of image data: BF only, SSC only, DNA only, SSC + DNA, BF + SSC, BF + DNA, and BF + SSC + DNA. Initially, the data were loaded into the software, and the appropriate input channels were selected. Immunophenotypically defined HSC and LSC populations were assigned as truth populations. Maximum image sizes were set to 150 × 150 pixels to ensure that no images were excluded based on size, and pixel values were normalized to the range 0 to 1 [78]. The AAI software then randomly partitioned the data into training, validation, and test sets by a preconfigured 80/10/10 ratio [68], with the goal of creating models with the highest accuracy possible when used to classify unseen data. While training was performed using the training data, accuracy was calculated following each iteration for both the training and validation sets, and the two metrics were followed during training by the generation of accuracy curves (Figure S6). Training was stopped upon convergence of training and validation accuracy, following a pre-configured algorithm within the AAI software. Lastly, model performance was evaluated by assessment of classification of the test data, which was unseen by the CNN during model training. For both LSC and HSC classes, the number of true positive (TP), true negative (TN), FP, and FN results were obtained. The following performance metrics were calculated: accuracy, recall, precision, and the F1 score. In general, accuracy delineates how often classification is correct by calculating the percentage of true predictions out of the total number of events:

$$Accuracy=\frac{TP+TN}{TP+TN+FP+FN}$$

Recall denotes the percentage of all positive events that were classified as positive by the model, also known as the true positive rate or sensitivity:

$$Recall=\frac{TP}{TP+FN}$$

Precision is defined as the proportion of positive events that were correctly classified:

$$Precision=\frac{TP}{TP+FP}$$

The F1 score represents the harmonic mean of precision and recall [66,68]:

$$F_{1}=\frac{2\cdot precision\cdot recall}{precision+recall}$$

These statistical measures were applied to analyze our data. The results were visualized by the creation of figures using RStudio (V1.3.1093, Posit, Boston, MA, USA).

### 4.8. Patient-Specific LSC Classification

A fraction of the CLEC12A^+^ LSCs gated from AML samples were retained and not utilized for CNN model development and testing due to class balancing of HSC-LSC data sets. Consequently, additional CLEC12A^+^ LSCs were available from the AML samples and utilized for further model evaluation on a per-patient basis. A total of 8487 CLEC12A^+^ LSCs were selected from AML patient no. 1 (*n* = 2700), 2 (*n* = 2667), 3 (*n* = 420), and 4 (*n* = 2700). All CLEC12A^+^ LSCs from AML patient no. 5 had already been included in previous analyses. Each of the seven models were employed for classification of these stem cells as either HSCs or LSCs. The percentage of predicted LSCs, corresponding to LSC recall, was determined both overall and separately for each AML patient.

## Figures and Tables

**Table 1:** Accuracy statistics.

Model Class	Training Data				Validation Data				Testing Data			
	Objects (*n*)	Precision (%)	Recall (%)	F1 (%)	Objects (*n*)	Precision (%)	Recall (%)	F1 (%)	Objects (*n*)	Precision (%)	Recall (%)	F1 (%)
**BF**												
*HSC*	7054	80.84	97.33	88.33	882	81.11	97.85	88.69	882	80.99	96.60	88.11
*LSC*	7054	96.65	76.94	85.67	882	97.29	77.21	86.09	882	95.79	77.32	85.57
*Weighted avg.*	14,108	88.75	87.13	87.00	1764	89.20	87.53	87.39	1764	88.39	86.96	86.84
**SSC**												
*HSC*	7054	61.58	64.66	63.08	882	61.42	65.87	63.57	882	61.36	64.63	62.95
*LSC*	7054	62.80	59.65	61.19	882	63.20	58.62	60.82	882	62.63	59.30	60.92
*Weighted avg.*	14,108	62.19	62.16	62.13	1764	62.31	62.24	62.20	1764	62.00	61.96	61.93
**DNA**												
*HSC*	7054	78.77	75.28	76.98	882	76.04	74.83	75.43	882	76.11	71.88	73.94
*LSC*	7054	76.33	79.71	77.98	882	75.22	76.42	75.82	882	73.36	77.44	75.34
*Weighted avg.*	14,108	77.55	77.50	77.48	1764	75.63	75.62	75.62	1764	74.74	74.66	74.64
**SSC + DNA**												
*HSC*	7054	80.84	82.04	81.43	882	78.42	81.97	80.16	882	79.17	79.71	79.44
*LSC*	7054	81.77	80.55	81.15	882	81.12	77.44	79.23	882	79.57	79.02	79.29
*Weighted avg.*	14,108	81.30	81.29	81.29	1764	79.77	79.71	79.69	1764	79.37	79.37	79.36
BF + SSC												
*HSC*	7054	81.87	95.11	87.99	882	82.43	95.24	88.37	882	81.59	94.44	87.55
*LSC*	7054	94.17	78.93	85.88	882	94.36	79.71	86.42	882	93.41	78.68	85.42
*Weighted avg.*	14,108	88.02	87.02	86.94	1764	88.40	87.47	87.40	1764	87.50	86.56	86.48
**BF + DNA**												
*HSC*	7054	93.41	96.31	94.84	882	94.14	96.49	95.30	882	91.72	95.46	93.56
*LSC*	7054	96.20	93.21	94.68	882	96.40	93.99	95.18	882	95.27	91.38	93.29
*Weighted avg.*	14,108	94.81	94.76	94.76	1764	95.27	95.24	95.24	1764	93.50	93.42	93.42
**BF + SSC + DNA**												
*HSC*	7054	94.88	94.78	94.83	882	95.24	95.24	95.24	882	93.70	92.74	93.22
*LSC*	7054	94.79	94.88	94.84	882	95.24	95.24	95.24	882	92.82	93.76	93.29
*Weighted avg.*	14,108	94.83	94.83	94.83	1764	95.24	95.24	95.24	1764	93.26	93.25	93.25

Seven different CNN classifiers were developed to discriminate healthy HSCs and aberrant LSCs using different combinations of BF, SSC, and DNA images captured on the ISX in Ch01, Ch06, and Ch07, respectively. The train-validation-test split technique was applied using a preconfigured 80/10/10 ratio. Model performance was assessed by calculation of precision, recall, and F1 scores for both the training, validation, and testing data. All metrics are presented for both HSC and LSC classes separately and combined. Abbreviations: BF; brightfield, Ch; channel; CNN, convolutional neural network; HSC, hematopoietic stem cell, ISX; ImageStream^®X^ Mark II, LSC; leukemic stem cell, SSC; side scatter.

**Table 2:** Patient characteristics.

Patient No.	1	2	3	4	5
**Age at diagnosis**	67	71	69	63	56
**Gender**	M	M	M	F	F
**Blast** percentage *	68%	20%	62%	94%	49%
**Percentage of CLEC12A^+^ LSCs ^†^**	30.42%	1.73%	0.09%	4.17%	0.09%
**Karyotype ^‡^**	46,XY [25]	Complex karyotype	46,XY,add(1)(q31) [4]/46,XY [21]	46,XX [25]	46,XX,inv(16)(p13q22) [23]/46,XX [2]
**iFISH ^§^**	No clonal aberrations	del(5q)(80%), del(7q)(72%), monosomi16 (78%)	ND	inv(16)(76%)	inv(16)(68%)
**Immunophenotype**	CD45^low^CD34^+^ CD117^+^CD13^+^ CD33^−^HLA-DR^+^ CD14^−^CD64^−^ CD38^−/(+)^CLEC12A^+^CD123^+^CD7^−^ CD19^−^CD10^−^	CD45^low^CD34^+^ CD117^+^CD38^+/−^CD13^+^HLA-DR^+^ CD33^+^CLEC12A^+/−^ CD123^(+)^CD38^+/−^	CD45^low^CD117^+/−^CD13^+^HLA-DR^+^ CD33^(+)^CD56^+^ CD38^+^CD7^−^CD19^−^CLEC12A^+^CD123^+/−^	CD45^low^CD34^+^ CD117^+^HLA-DR^+^ CD13^+^CD33^+^ CD123^+^CLEC12A^+^ CD64^−^CD38^+/−^	CD34^+^CD117^+^ CD13^+^CD33^+^ HLA-DR^+^CD38^+^ CD64^+^CLEC12A^+^ CD123^+^CD14^−^CD4^−^CD56^−^ CD2^+/−^
***FLT3*** **-ITD**	No mutation	No mutation	Mutation	No mutation	No mutation
**Targeted panel** **sequencing**	*ASXL1*, *CEBPA*(x3), *NRAS*(x2), *SRSF2*, *TET2*(x2)	*TP53*	*ASXL1*, *EZH2*(x2), *FLT3*(ITDx2, TKDx1), *RUNX1*, *SETBP1*	*ASXL1*, *FLT3* (TKDx2)	No mutations

* The fraction of blasts was concluded from cytological examination. **^†^** Determined from investigating routine diagnostic flow cytometric data (Figure S2) **^‡^** Based on analysis of 25 metaphases. **^§^** iFISH was performed using probes targeting del(5q), del(7q), t(8;21), and inv(16). Abbreviations: F, female; iFISH, interphase fluorescence in situ hybridization; ITD, internal tandem duplication; M, male; ND, not done; TKD, tyrosine kinase domain.

**Table 3:** Number of HSCs and LSCs gated from each NBM and AML sample.

NBM	No. HSCs	AML	No. LSCs
NBM1	1543	AML1	1840
NBM2	547		
NBM3	299	AML2	1840
NBM4	1393		
NBM5	522	AML3	1839
NBM6	1285		
NBM7	96	AML4	1840
NBM8	584		
NBM9	861	AML5	1459
NBM10	1688		

All 8818 HSCs available from the NBM samples were included in the dataset. Class balancing was performed by reducing the number of LSCs gated from AML samples. Among the 44,277 available LSCs, a total of 8818 LSCs were utilized. These were divided equally among the AML samples—except AML5, which contained a total of 1459 LSCs—and randomly selected from each patient-specific LSC pool.

## Data Availability

The data presented in this study are available on reasonable request from corresponding author M.L.

## Supplementary Materials

The following supporting information can be downloaded at: https://www.mdpi.com/article/10.3390/ijms25126465/s1.
