# Supplementary material for: Imaging Flow Cytometry and Convolutional Neural Network-Based Classification Enable Discrimination of Hematopoietic and Leukemic Stem Cells in Acute Myeloid Leukemia
Source: Int J Mol Sci. 2024 Jun 12;25(12):6465. doi: 10.3390/ijms25126465 (PMC11203419; doi:10.3390/ijms25126465)
Supplement: Supplementary file 1 [file ijms-25-06465-s001.zip › Supplementary Data.pdf]

Supplementary Data

Imaging Flow Cytometry and Convolutional Neural Network-Based Classification Enable Discrimination of Hematopoietic and Leukemic Stem Cells in Acute Myeloid Leukemia

Hybel et al. 2024, International Journal of Molecular Sciences

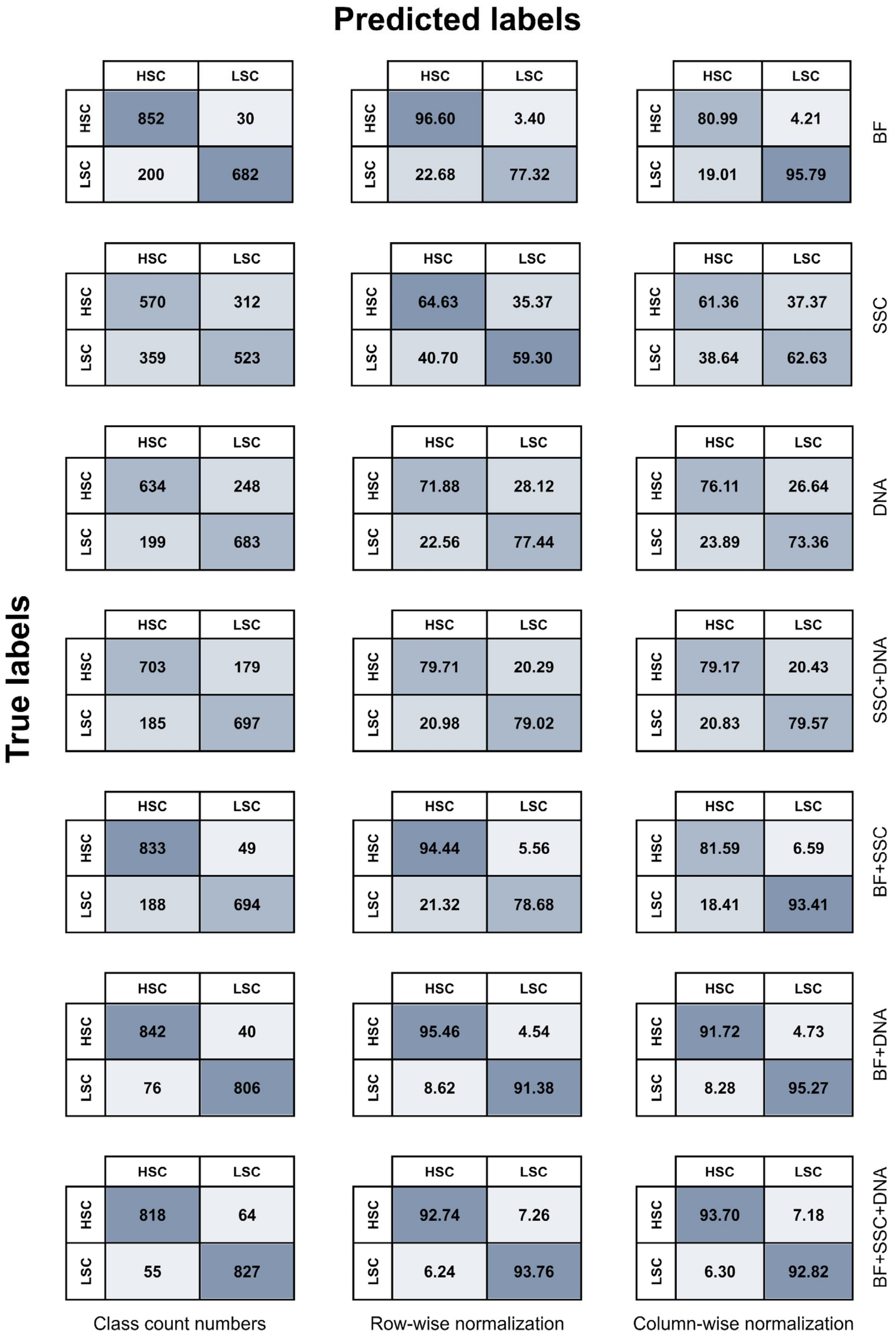

Figure S1. Confusion matrices. All stem cells in the test data (n=1,764) were classified as HSCs or LSCs using each of the seven developed AI models based on BF, SSC, DNA, SSC+DNA, BF+SSC, BF+DNA, or

BF+SSC+DNA images. For each model, the relationship between true and predicted labels are shown as class count numbers, specifying TP, TN, FP, and FN events (left). Row-wise normalization was conducted, delineating recall/sensitivity for each category (middle). Colum-wise normalization was applied, showing precision for each category (right). Abbreviations: BF; brightfield, FN; false negative, FP; false positive, HSC; hematopoietic stem cell, LSC; leukemic stem cell, SSC; side scatter, TN; true negative, TP; true positive.

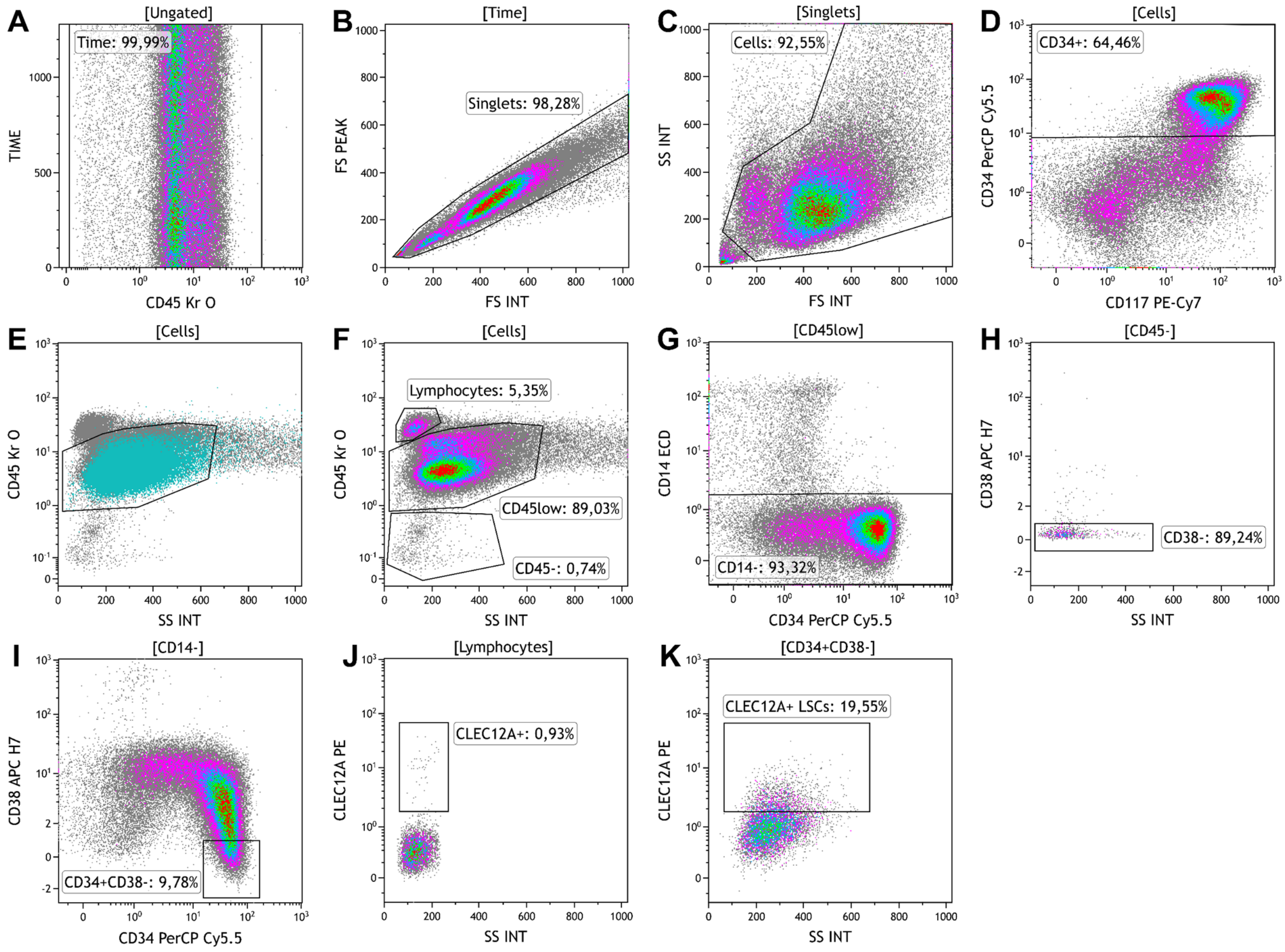

Figure S2. Frequency of CLEC12A⁺ LSCs in AML BM samples. Example showing the gating procedure used to determine the frequency of CLEC12A⁺ LSCs within AML samples. Analyses were performed using data files previously acquired during routine diagnostic procedures at the Department of Hematology, Aarhus University Hospital, Denmark. The results were used to determine the eligibility for the current study as one of the inclusion criteria was ≥0.025% CLEC12A⁺ LSCs out of the total number of events acquired. **(A)** Initially, cells in stable flow were selected using the time parameter. **(B)** Single cells were gated in a bivariate plot of FS INT and FS PEAK. **(C)** Debris was removed based on having low FS INT and low SS INT, and dead cells were excluded as those having low FSC INT combined with high SSC INT. **(D)** Next, CD34⁺ blasts were identified and **(E)** shown in blue in a bivariate plot of CD45 and SS INT. **(F)** Based on this as well as CD45 expression levels and granularity, three populations were identified: (i) lymphocytes being CD45^{high}SS^{low}, (ii) CD45⁻ cells including immature erythroblasts, and (iii) a CD45^{low} population containing blasts, HSCs, LSCs, monocytes,

plasma cells, and SS^{low} granulocytes, although excluding SS^{high} granulocytes. **(G)** CD14^{+} monocytes were excluded from the CD45^{low} cells. **(H)** Erythroblasts were used as an internal negative control to set the boundary for CD38 positivity. **(I)** Finally, the CD34^{+}CD38^{-} stem cell compartment was identified in a CD34/CD38 bivariate plot. **(J)** Lymphocytes gated in (F) were used as an internal negative control to set the boundary for the positivity of CLEC12A. **(K)** Among CD34^{+}CD38^{-} cells, LSCs expressing aberrant marker CLEC12A were identified by placing a gate based on the threshold established in (J). Analyses were performed using Kaluza (V1.3, Beckman Coulter). Abbreviations: AML; acute myeloid leukemia, BM; bone marrow, FS; forward scatter, HSC; hematopoietic stem cell, LSC; leukemic stem cell, SS; side scatter.

Table S1. Antibodies and dyes included in the HSC-LSC panel.

Marker	Fluorochrome/dye	Clone	Company	Cat #	Volume* (μL/100μL)
**Viability**	Zombie Green	NA	BioLegend	423111	0.125, 0.25, 0.5†
**CLEC12A**	PE	HB3	Created in-house and conjugated by Agilent	NA	8
**CD14**	PE-Texas Red	Tuk4	ThermoFisher Scientific	MHCD1417	0.156
**DNA**	Vybrant Dyecycle Violet	NA	Invitrogen	V35003	1.5 (uM)
**CD45**	Krome Orange	J33	Beckman Coulter	B36294	2.5
**CD38**	StarBright Violet 610	AT13/5	Bio-Rad	MCA1019SBV610	2.5, 5†
**CD34**	Alexa Flour 647	581	BioLegend	343508	1.25

*Volumes are specified as μL antibody/dye added per 100 μL total solution containing $2{\cdot}10^{6}$ cells. †Stain and antibody were re-titrated upon changing lot #.

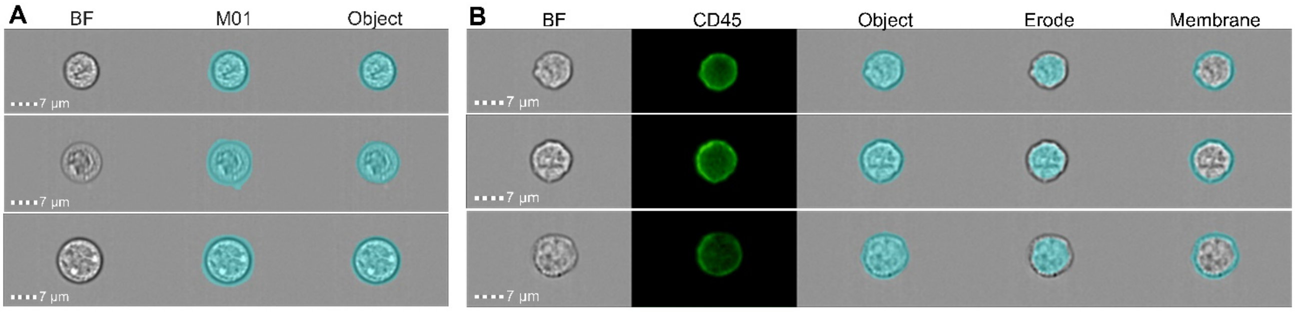

Figure S3. CD45 KrO masking strategy. A mask covering the pixels corresponding to the cell membrane was created and utilized for calculation of CD45 KrO intensity. **(A)** Initially, an object mask was created based on Ch01 BF images (right column), providing better selection of the cell area when compared with the default M01 mask (middle column). **(B)** The erode function was used on the object mask to exclude the four outermost pixels, which resulted in selection of the intracellular area (second-most right column). By subtracting the eroded mask from the object mask, only pixels corresponding to the cell membrane were masked (right column). Masks are shown in blue. Abbreviations: BF; brightfield, Ch; channel.

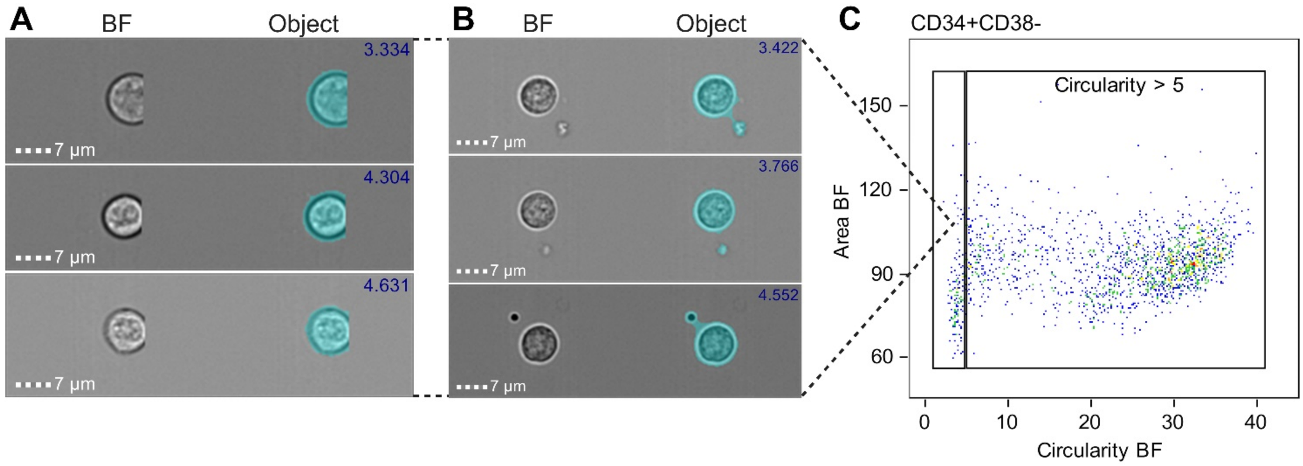

Figure S4. Image clean-up. Following immunophenotypic gating of HSCs and LSCs, a strategy was developed for automatic removal of any undesirable images that otherwise may confound the AI model, including **(A)** clipped cell images and **(B)** images containing debris fragments along with the cell. Based on Ch01 BF images (left columns), an object mask was created (right columns) and used to calculate a circularity feature. The circularity feature value is indicated for each cell in the upper right corner. Manual inspection of images revealed an optimal circularity limit of 5. **(C)** Images with a circularity feature >5 were gated and used for subsequent model training. Masks are shown in blue. Abbreviations: AI; artificial intelligence, BF; brightfield, Ch; channel, HSC; hematopoietic stem cell, LSC; leukemic stem cell.

A

CELLULAR REGION			
**Mask name**	**Description**	**BF**	**Mask**
M01, Ch01 BF	Default mask capturing all light detected in the images. Highlights the cellular region in an inclusive manner.		
Object(M01, Ch01 BF, Tight)	Identification of the cell area by detecting all pixels that deviate from background pixels, providing a tighter fit compared with the default cell mask.		

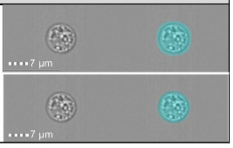

B

NUCLEUS				
**Mask name**	**Description**	**BF**	**VDCV**	**Mask**
M07, Ch07 VDCV	Default channel mask capturing all detected light. Used for selection of the nuclear area of each cell.			

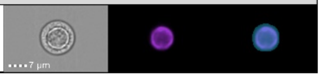

C

CYTOPLASM			
**Mask name**	**Description**	**BF**	**Mask**
Erode(Object(M01, Ch01 BF, Tight), 4)	Selection of the cytoplasmic area by subtraction of the outermost four pixels corresponding to the cell membrane.		

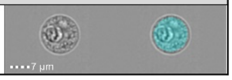

D

MEMBRANE REGION			
**Mask name**	**Description**	**BF**	**Mask**
Object(M01, Ch01 BF, Tight) And Not Erode(Object(M01, Ch01 BF, Tight), 4)	Exclusion of the cytoplasmic region from the object mask covering the cellular area, thereby ensuring selection of the cell membrane.		

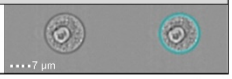

E

FLUORESCENCE				
**Mask name**	**Description**	**BF**	**Fluorescence**	**Mask**
M02, Ch02 ZG	Default channel masks capturing the light detected in the images. Used to mask the fluorescence detected from each fluorophore/dye in an inclusive manner.			
M03, Ch03 CLEC12A PE				
M04, Ch04 CD14 PE-Texas Red				
M10, Ch10 CD38 SBV610				
M11, Ch11 CD34 AF647				

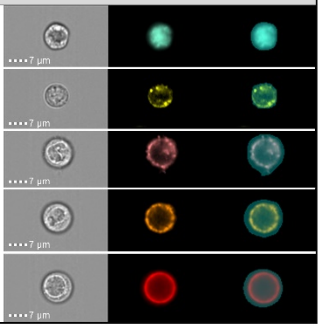

F

GRANULARITY				
**Mask name**	**Description**	**BF**	**SSC**	**Mask**
M06, Ch06 SSC	Default channel mask capturing all detected light. Highlights SSC signals and thus granular areas of each cell.			

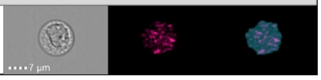

Figure S5. Masking. Detailed descriptions of all masks used during the gating strategy performed to identify LSCs and HSCs. Masks were used to highlight regions of interest within images. **(A)** The entire cellular region was selected, both inclusively using the default M01 mask and restrictively by applying the object mask providing a tighter fit. **(B)** Nuclear cell areas were captured using the default M07 mask to highlight light detected in Ch07, corresponding to signals from the DNA dye VDCV. **(C)** The cell cytoplasm was masked using the erode function on the object mask, subtracting the outermost four pixels. **(D)** The mask marking the cellular cytoplasm was subtracted from the object mask, thereby resulting in selection of the membrane region. **(E)** Fluorescence signals were captured using default masks that highlight all detected light in the specified fluorescence channels. **(F)** Granular cell areas were masked by selecting areas where SSC light signals were detected within Ch06. Representative images are displayed on the right, showing BF images (first column), fluorescence images (middle column, where relevant), and the described mask (last column). Masks are shown in blue. Abbreviations: BF; brightfield, Ch; channel, HSC; hematopoietic stem cell, LSC; leukemic stem cell, SSC; side scatter, VDCV; vybrant dyecycle violet, ZG; zombie green.

Table S2. Feature list.

Category	Feature	Definition	Usage
**Signal strength**	Intensity	Sum of the pixel values within the selected mask with the background subtracted.	Separation of cell populations that are either positive or negative for a specific marker.
	Raw Max Pixel	Largest value of pixels within mask.	Removal of objects with signals that are out of range.
	Saturation Count	Number of saturated pixels counted within the image of the selected object.	Removal of object images containing one or more saturated pixels.
**Size**	Area	Number of μm^2 within the selected mask.	Separation of singlets from aggregates of cells and debris.
**Texture**	Gradient Root Mean Square	A measure of image sharpness quality. Detects the average gradient of a pixel normalized using intensity level variations.	Gating of images in focus.
**Shape**	Aspect Ratio	Measures the roundness of an object by dividing the minor axis by the major axis of the object mask.	Separation of singlets from aggregates of cells and debris.
	Circularity	Determines the variation from a circle by calculating the average distance between the center and the boundary of a mask divided by the distance variation.	Removal of clipped images or images containing debris along with the cell.
**System**	Time	The camera timer values converted to seconds.	Gating of cells in stable flow.

List of features used in the series of gating steps leading to identification of HSCs and LSCs. The various features are categorized, and feature definitions and specific usage in the current study are described. All feature definitions are originally described in the IDEAS user manual**.** Abbreviations: HSC; hematopoietic stem cell, LSC; leukemic stem cell.

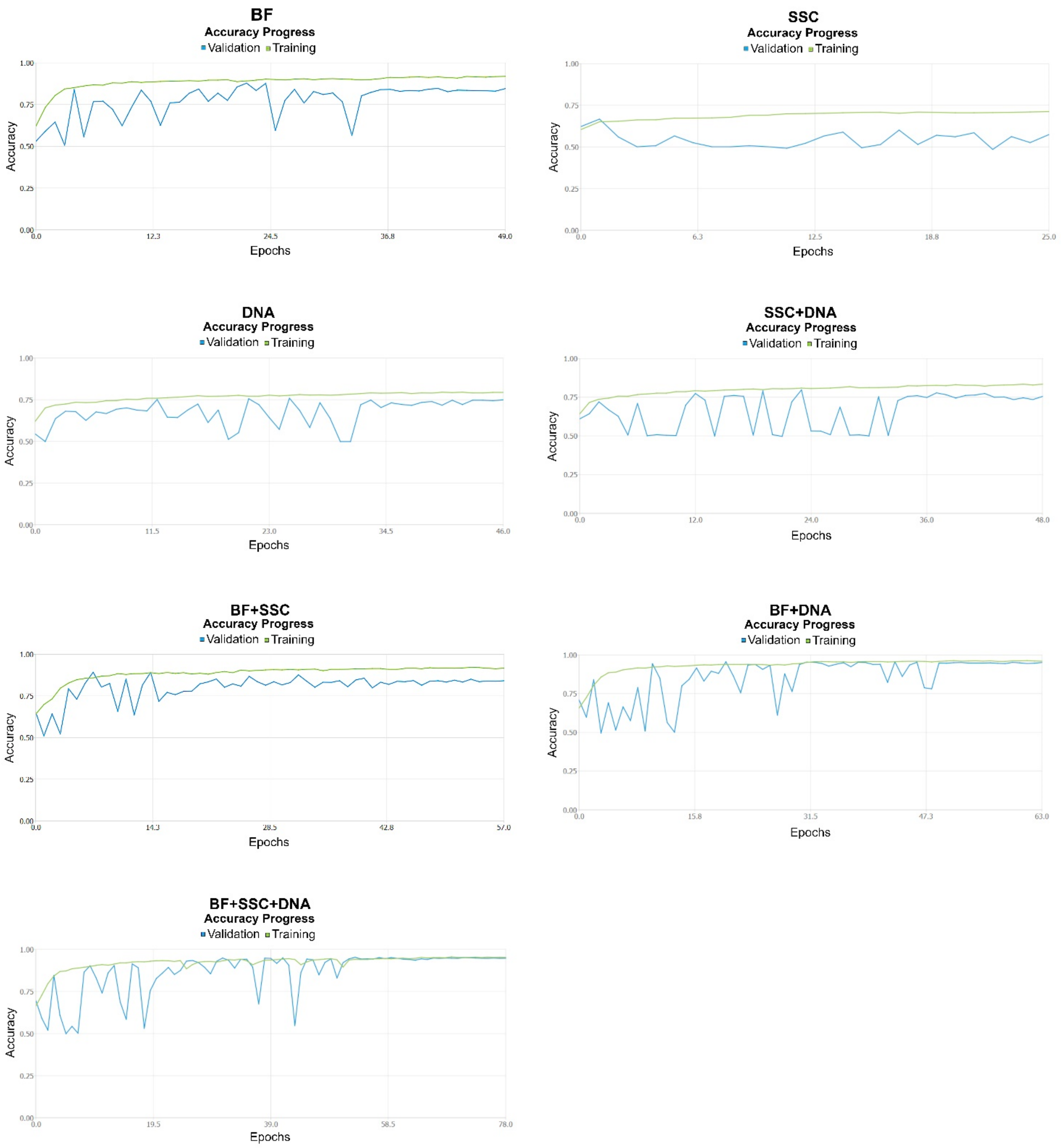

Figure S6. Accuracy curves. Seven CNN classifiers were trained to discriminate HSCs and LSCs based on different combinations of BF, SSC, and DNA images using the AAI software. Accuracy was calculated for both the training and validation data following each epoch and plotted. Accuracy curves are presented for each AI model, showing accuracy progress during training and the number of epochs. Training was stopped upon convergence of training and validation accuracy, following a pre-configured algorithm within the AAI software. Abbreviations: AI; artificial intelligence, AAI; Amnis AI, BF; brightfield, CNN; convolutional neural network, HSC; hematopoietic stem cell, LSC; leukemic stem cell, SSC; side scatter.
