# Supplementary material for: Imaging Flow Cytometry and Convolutional Neural Network-Based Classification Enable Discrimination of Hematopoietic and Leukemic Stem Cells in Acute Myeloid Leukemia
Source: Int J Mol Sci. 2024 Jun 12;25(12):6465. doi: 10.3390/ijms25126465 (PMC11203419; doi:10.3390/ijms25126465)
Supplement: Supplementary file 1 [file ijms-25-06465-s001.zip › Table S1.pdf]

Table S1. Antibodies and dyes included in the HSC-LSC panel.

Marker	Fluorochrome/dye	Clone	Company	Cat #	Volume* (μL/100μL)
**Viability**	Zombie Green	NA	BioLegend	423111	0.125, 0.25, 0.5ᵻ
**CLEC12A**	PE	HB3	Created in-house and conjugated by Agilent	NA	8
**CD14**	PE-Texas Red	Tuk4	ThermoFisher Scientific	MHCD1417	0.156
**DNA**	Vybrant Dyecycle Violet	NA	Invitrogen	V35003	1.5 (uM)
**CD45**	Krome Orange	J33	Beckman Coulter	B36294	2.5
**CD38**	StarBright Violet 610	AT13/5	Bio-Rad	MCA1019SBV610	2.5, 5ᵻ
**CD34**	Alexa Flour 647	581	BioLegend	343508	1.25

*Volumes are specified as μL antibody/dye added per 100 μL total solution containing $2 \cdot 10^6$ cells. ᵻStain and antibody were re-titrated upon changing lot #.
