# Supplementary material for: Imaging Flow Cytometry and Convolutional Neural Network-Based Classification Enable Discrimination of Hematopoietic and Leukemic Stem Cells in Acute Myeloid Leukemia
Source: Int J Mol Sci. 2024 Jun 12;25(12):6465. doi: 10.3390/ijms25126465 (PMC11203419; doi:10.3390/ijms25126465)
Supplement: Supplementary file 1 [file ijms-25-06465-s001.zip › Table S2.pdf]

**Table S2.** Feature list.

| Category | Feature | Definition | Usage |
|---|---|---|---|
| **Signal strength** | Intensity | Sum of the pixel values within the selected mask with the background subtracted. | Separation of cell populations that are either positive or negative for a specific marker. |
| | Raw Max Pixel | Largest value of pixels within mask. | Removal of objects with signals that are out of range. |
| | Saturation Count | Number of saturated pixels counted within the image of the selected object. | Removal of object images containing one or more saturated pixels. |
| **Size** | Area | Number of $\mu m^2$ within the selected mask. | Separation of singlets from aggregates of cells and debris. |
| **Texture** | Gradient Root Mean Square | A measure of image sharpness quality. Detects the average gradient of a pixel normalized using intensity level variations. | Gating of images in focus. |
| **Shape** | Aspect Ratio | Measures the roundness of an object by dividing the minor axis by the major axis of the object mask. | Separation of singlets from aggregates of cells and debris. |
| | Circularity | Determines the variation from a circle by calculating the average distance between the center and the boundary of a mask divided by the distance variation. | Removal of clipped images or images containing debris along with the cell. |
| **System** | Time | The camera timer values converted to seconds. | Gating of cells in stable flow. |

List of features used in the series of gating steps leading to identification of HSCs and LSCs. The various features are categorized, and feature definitions and specific usage in the current study are described. All feature definitions are originally described in the IDEAS user manual**.** Abbreviations: HSC; hematopoietic stem cell, LSC; leukemic stem cell.
